# Liver CT-based composite biomarkers can identify MASH and steatosis grade in people with obesity prior to bariatric surgery: a retrospective study

**DOI:** 10.1186/s12876-026-04914-2

**Published:** 2026-05-14

**Authors:** Hailong Zhang, Zhuoru Jiang, Huanhuan Zheng, Haoyao Wang, Liankun Xia, Jun Chen, Bing Zhang

**Affiliations:** Department of Radiology, Nanjing Drum Tower Hospital, Affiliated Hospital of Medical School, Nanjing University, No. 321, Zhongshan Road, Nanjing, Jiangsu 210008 China

**Keywords:** Obesity, Metabolic Dysfunction-associated Steatohepatitis, Hepatic Steatosis, Computed Tomography, Chinese patients

## Abstract

**Background:**

Hepatic steatosis is a precursor to metabolic dysfunction-associated steatohepatitis (MASH), which is linked to obesity and diabetes and poses a risk for liver cirrhosis and cancer. For detecting MASH and hepatic steatosis, liver biopsy is the reference method, but it is invasive and impractical for repeated monitoring of treatment efficacy. Our goal was to develop a convenient and non-invasive tool for accurately identifying MASH and steatosis grade in people with obesity.

**Methods:**

This retrospective study included 321 people with obesity who underwent bariatric surgery, during which liver biopsies were also performed. Clinical characteristics, presurgical computed tomography (CT)-derived parameters, magnetic resonance imaging proton density fat fraction (MRI-PDFF), and CT-based skeletal muscle index (SMI) were obtained.

**Results:**

A convenient model consisting of liver CT attenuation value (CT_Liver_), alanine aminotransferase (ALT) and high-density lipoprotein cholesterol (HDL-C) was developed for identifying MASH, with area under the curve (AUC) of 0.799. To predict hepatic steatosis of grade ≥ 1, ≥ 2 and 3, the AUCs of CT_Liver_-based combination models were 0.954, 0.927, and 0.894, respectively. The diagnostic performance of the CT_Liver_-based combination models was comparable to that of liver MRI-PDFF in identifying different hepatic steatosis grades (Delong’s test, *p* > 0.05). CT-based SMI was significantly positively correlated with hepatic steatosis grade (*r* = 0.12, *p* = 0.031) and non-alcoholic fatty liver disease (NAFLD) activity score (*r* = 0.18, *p* = 0.002).

**Conclusions:**

The liver CT-based composite biomarkers can identify MASH and hepatic steatosis grade with high diagnostic performance in people with obesity, providing a non-invasive, accessible, and practical supplementary tool for clinical stratified management.

**Supplementary Information:**

The online version contains supplementary material available at 10.1186/s12876-026-04914-2.

## Introduction

With the global epidemic of type 2 diabetes mellitus (T2DM), obesity and metabolic syndrome, half of the population with overweight/obesity now have metabolic dysfunction-associated steatotic liver disease (MASLD) [1]. MASLD was previously known as non-alcoholic fatty liver disease (NAFLD), with a spectrum from simple steatosis, metabolic dysfunction-associated steatohepatitis (MASH), liver fibrosis, to cirrhosis, and eventually MASH-related hepatocellular carcinoma (HCC) [2]. Globally, the prevalence of MASH in the general population is estimated at approximately 5.27% [3], with a moderately increased prevalence of 6.7% reported in the adult population of China [4]; of note, the prevalence rate of MASH among individuals with T2DM is as high as 31.55% [5]. If detected, simple steatosis and MASH can be treated with early clinical intervention; therefore, early identification of steatosis and MASH is of critical importance in the management of MASLD and improving patients’ long-term outcomes.

Liver biopsy remains the reference standard for diagnosing hepatic steatosis and MASH. This invasive procedure has several notable limitations, such as procedural pain, bleeding, infection, sampling error, and inter-observer diagnostic variability [6], which restrict its broad application in clinical practice. Currently, non-invasive imaging techniques for diagnosing hepatic steatosis mainly include ultrasound, computed tomography (CT) and magnetic resonance imaging proton density fat fraction (MRI-PDFF). Conventional ultrasound is widely available and frequently employed as the initial evaluation in patients with elevated serum liver enzyme levels and suspected hepatic steatosis. However, it is highly dependent on the operator experience and the platform and has poor sensitivity in the diagnosis of steatosis in people with morbid obesity [7]. Ultrasound-based controlled attenuation parameter (CAP) measured by FibroScan device is also a well-validated, widely used non-invasive tool for hepatic steatosis assessment. However, CAP diagnostic cutoffs for steatosis grading vary substantially by liver disease etiology, and CAP values are found to be independently impacted by clinical confounding factors including etiology, diabetes, body mass index (BMI), aspartate aminotransferase (AST) and sex [8]. Furthermore, CAP tends to overestimate hepatic steatosis severity, limiting its clinical utility in populations with obesity [9]. MRI has been proven to be an excellent non-invasive imaging modality for diagnosing hepatic steatosis and steatohepatitis [10, 11]. Nevertheless, it should be noted that MRI is not widely utilized in routine clinical practice at present due to the relatively high cost and low availability.

Obesity can be stratified into two distinct clinical phenotypes: metabolically healthy obesity (MHO) and metabolically unhealthy obesity (MUO). Emerging evidence has identified MASLD as a critical, independent predictor of the phenotypic conversion from MHO to MUO, as well as a risk factor for major adverse cardiovascular events in individuals with MHO. These findings highlight that the assessment of hepatic health is of critical importance in the clinical evaluation for MHO [12]. Obesity and MASLD are frequently concurrent, but they do not necessarily share an identical pathogenic mechanism. Recent studies using human hepatocytes have demonstrated that weight loss following the correction of a hypercaloric diet is not fully aligned with the resolution of hepatic steatosis and steatohepatitis. The inflammatory regulatory effect of weight loss varies significantly depending on the background of patatin-like phospholipase domain-containing 3 [13, 14]. Despite the well-established epidemiological association between obesity and MASLD, this association is mainly attributed to the complexity of human dietary components, which can simultaneously drive the development of hepatic steatosis and steatohepatitis. Lifestyle interventions including a healthy diet and increased physical activity remain the cornerstone of clinical management for MASLD and obesity to date. Furthermore, bariatric surgery typically yields more substantial therapeutic benefits for MASLD patients with obesity. This surgical intervention can mitigate liver inflammation and fibrosis at the histological level and reduce the risk of T2DM-related macrovascular complications [15, 16].

In our hospital, prior to bariatric surgery, patients are scheduled to undergo non-contrast chest CT to rule out cardiovascular and lung diseases as part of surgical planning. The superior-inferior range of the chest CT is extensive enough to capture at least part of the liver, often revealing hepatic steatosis incidentally. It is well known that CT-based liver attenuation and liver-spleen attenuation ratio can serve as imaging markers for diagnosing hepatic steatosis, although its efficacy varies across different reports [17–19]. A few studies have explored the capabilities of dedicated abdominal CT, which is not widely available prior to the bariatric surgery for detecting MASH [20, 21]. Chest CT is widely used in clinical practice for cardiovascular and pulmonary disease screening, including routine preoperative evaluation prior to bariatric surgery. To the best of our knowledge, few studies, with liver biopsy available, have investigated its performance in detecting MASH in individuals with obesity. Therefore, we aimed to develop a convenient, accurate and non-invasive method using the chest CT for identifying MASH and steatosis grade in patients with obesity prior to surgery.

## Methods

### Subjects

This study was conducted in accordance with the requirements of the Declaration of Helsinki and was approved by the Institutional Review Board (IRB, 2024-081-02) of Nanjing Drum Tower Hospital, Affiliated Hospital of Medical School, Nanjing University. Ethical approval for this study has waived the requirement for informed consent from participants.

**Fig. 1:**
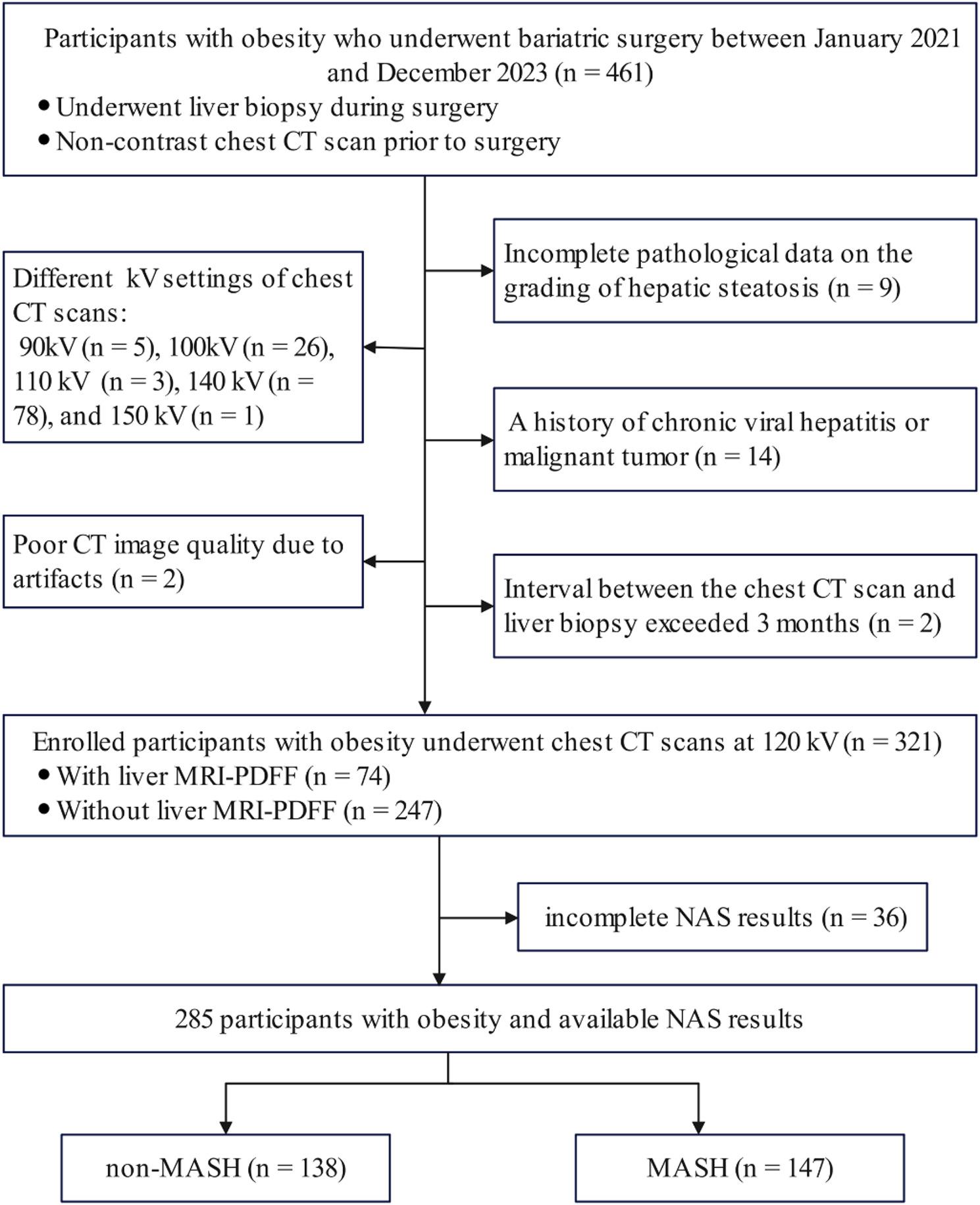
The flowchart of the participants inclusion and exclusion in this study. Kilovolts, kV. NAS, NAFLD activity score. MASH, metabolic dysfunction-associated steatohepatitis.

This study included participants with obesity who underwent bariatric surgery between January 2021 and December 2023. Patients who underwent liver biopsy simultaneously during surgery and non-contrast chest CT scan prior to surgery were eligible. It was important to note that different tube voltage settings in kilovolts (kV) affected CT attenuation measurement, and only 120 kV chest CT scans were included in this study. The exclusion criteria were as follows: (1) incomplete pathological data on the grading of hepatic steatosis; (2) a history of chronic viral hepatitis or malignant tumor; (3) presurgical chest CT scans with non-120 kV settings; (4) poor CT image quality due to artifacts; (5) the interval between the chest CT scan and liver biopsy exceeded 3 months (Fig. 1).

Finally, 321 patients with obesity who underwent bariatric surgery and presurgical chest CT (110 men and 211 women; mean age, 31.3 ± 9.3 years; range, 13–64 years) were included in the study, among which, 74 patients also underwent liver MRI-PDFF prior to the surgery.

### Clinical characteristics

Clinical characteristics for each patient within one week before the bariatric surgery were collected from the medical records in our hospital, including age, gender, BMI, hypertension, abnormal glucose metabolism (AGM) including prediabetes and T2DM, platelet (PLT), AST, alanine aminotransferase (ALT), total bilirubin (TBil), direct bilirubin (DBil), fasting glucose, triglycerides (TG), total cholesterol (TC), high-density lipoprotein cholesterol (HDL-C), low-density lipoprotein cholesterol (LDL-C), and C-reactive protein (CRP).

### CT and MR imaging protocol

All presurgical non-contrast chest CT images were acquired during inspiratory breath-hold with multidetector-row CT scanners manufactured by GE Healthcare (Chicago, IL, USA), Philips Healthcare (Amsterdam, Netherlands), Siemens Healthineers (Erlangen, Germany), and United Imaging Healthcare (Shanghai, China). All chest CT scans were acquired using a standardized acquisition protocol, the parameters of which were as follows: slice thickness, 5 mm; slice interval, 5 mm; tube voltage, 120 kV.

Liver MRI-PDFF images were obtained by two 3.0-Tesla MR scanners (Ingenia CX, Philips; uMR790, United Imaging). The time interval between MR scan and bariatric surgery was not more than one week. Prior to undergoing MRI-PDFF examination, patients must maintain a fasting state for at least 4 h. The MR sequences for liver fat quantification included mDixon Quant (Philips, Netherlands), and fat analysis calculation technique (FACT) (United Imaging, China). Imaging parameters of the MR sequences (mDixon Quant and FACT) were as follows: repetition time, 5.8/10.64 ms; echo time, 1.02/1.26 ms; field of view, 420 × 345 × 201/400 × 300 × 184 mm³; flip angle, 3°/3°; section thickness, 6/8 mm; number of echoes, 6/6; respectively.

### Analysis of CT-derived parameters and liver PDFF

Two radiologists (HH.Z, HL.Z.), with 7 and 10 years of experience in abdominal imaging, independently analyzed quantitative chest CT and liver MRI images and were blinded to clinicopathological information of the enrolled patients. 30 patients were randomly selected for repeated measurements to determine the inter-observer accuracy of CT and MRI quantitative parameters.

**Fig. 2:**
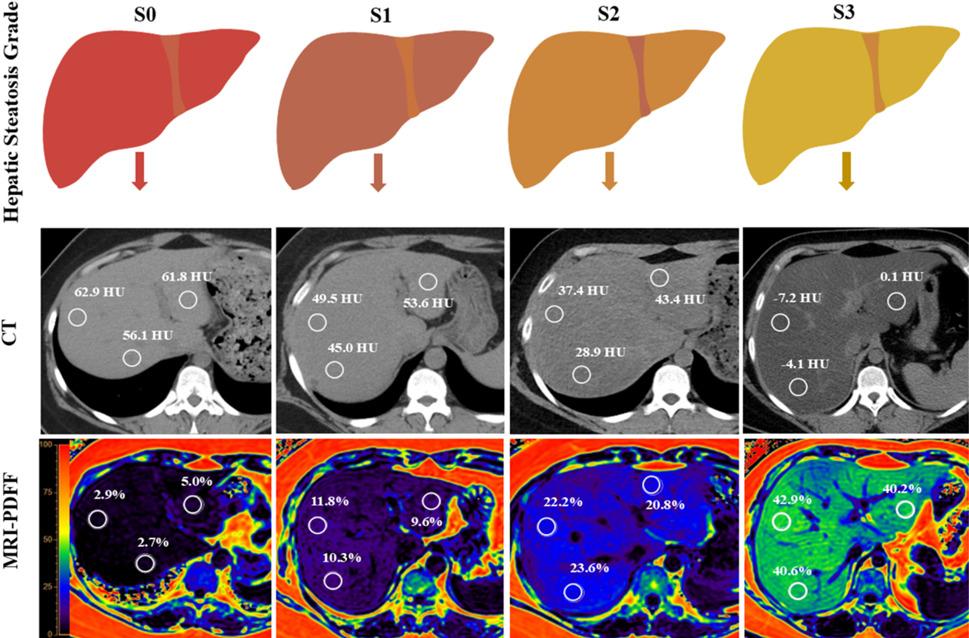
Non-contrast chest CT and liver MRI-PDFF images of pathologically confirmed different hepatic steatosis grades (S0 - 3). Three round ROIs were placed at the left lobe, anterior and posterior part of the liver right lobe, and the mean liver attenuation and PDFF value of the three ROIs were taken as the liver fat content.

After CT images acquisition, liver CT attenuation value (CT_Liver_) in Hounsfield Unit (HU) was measured by using Radiant DICOM Viewer software (version 2024.1; Medixant, Poznan, Poland). Regions of interest (ROIs) were placed at the transverse section where the left branch of portal vein appeared in the hepatic fissure. Three ROIs of the same size (300 mm^2^, deviation < 10 mm^2^) were manually placed at the peripheral region of left lobe, the anterior and posterior part of the right lobe, respectively. The ROIs were chosen to avoid the liver edge, intrahepatic lesions, grossly visible large blood vessels and bile ducts, as well as the area of artifacts. The post-processing of MRI data was performed by the software equipped with the MR scanners. On liver fat fraction maps, the sizes and locations of the three ROIs were manually matched with the chest CT images as closely as possible (Fig. 2). The average values of CT_Liver_ and PDFF within the three ROIs were used as the final measurement result.

For spleen, one circular ROI (300–310 mm²) was positioned in the central portion of the spleen, at the same transverse section where CT_Liver_ was measured. If the spleen was not visible or its cross-sectional area was less than 300 mm^2^, then the thickest splenic section shall be selected for measurement. The liver to spleen CT attenuation ratio (CT_L/S_) was then calculated.

### CT-based body composition-skeletal muscle index acquisition

A single axial CT image at the level of 11th or 12th thoracic vertebral body (T11 or T12) was selected, and the skeletal muscles at this level were semi-automatically segmented using predefined CT attenuation thresholds (− 29 to 150 HU) by ImageJ software (https://imagej.net/ij/index.html). The skeletal muscle index (SMI) in centimeters squared per meters squared was calculated by dividing the cross-sectional areas of the skeletal muscles by the patient’s height squared [22].

### Pathological diagnosis of liver biopsy tissue specimen

Pathological diagnosis was made based on the Nonalcoholic Steatohepatitis Clinical Research Network scoring system [23]. Pathologically, the grading criteria for hepatic steatosis according to the percentage of hepatocytes with macrovesicular or macrovesicular-predominant steatosis were as follows: none (< 5%, S0), mild (5% − 33%, S1), moderate (34% − 66%, S2), and severe (> 66%, S3). The sum of the scores for hepatocyte steatosis (0–3), hepatocyte ballooning (0–2) and lobular inflammation (0–3) was defined as the NAFLD activity score (NAS). Participants with a diagnosis of MASLD were further divided into MASH group as NAS ≥ 4 (with at least one point each for steatosis, ballooning and lobular inflammation), which was referred to a previous study [24].

### Statistical analysis

The Shapiro-Wilk test was used to check the normality assumption for all parameters. Since some parameters did not follow normal distribution (Supplementary File 1), all continuous variables were represented by medians and interquartile. Categorical variables were expressed as counts and percentages. Mann-Whitney U test was used to analyze continuous variables. The Chi-square test was used to compare categorical variables. Variables with *p* value < 0.05 in the univariate analysis were finally included in the multivariable analysis base on binary logistic regression by a backward stepwise. The prediction model and nomogram were then developed. The diagnostic performance of models in predicting hepatic steatosis grade and MASH was tested with receiver operating characteristic (ROC) curve analysis. DeLong’s test was used to compare the area under the curve (AUC). The relationships between CT-derived results, hepatic steatosis grades, and NAS were evaluated using Spearman’s correlation analysis. To assess the interobserver agreement, the intraclass correlation coefflcient (ICC) was calculated separately for liver MRI-PDFF, CT liver and spleen attenuation value. Statistical analysis was performed with SPSS (v.22.0 for Microsoft Windows x64, SPSS, Chicago, IL). ROC analysis was performed by MedCalc Statistical Software version 19 (MedCalc Software bvba, Ostend, Belgium; https://www.medcalc.org; 2019). A nomogram for predicting MASH risk was conducted with the assistance of R software (version 4.5.0).

## Results

### Basic characteristics

The 321 participants had a mean BMI of 38.1 kg/m^2^. There were 32 (10.0%) participants with hepatic steatosis grade 0, 86 (26.8%) with grade 1, 115 (35.8%) with grade 2, and 88 (27.4%) with grade 3. 36 patients had incomplete NAS results in the liver biopsy pathological records and were excluded from the subsequent statistical analysis related to MASH. Among the other 285 patients with available NAS results, the scores were distributed as follows: NAS 1 (12, 4.2%), NAS 2 (36, 12.6%), NAS 3 (70, 24.6%), NAS 4 (88, 30.9%), NAS 5 (64, 22.5%), and NAS 6 (15, 5.3%). Based on the pathological diagnostic criteria, 138 cases (48.4%) were classified as non-MASH and 147 cases (51.6%) as MASH.

### Differences between clinical-CT parameters and MASH, steatosis

#### MASH

Table 1 showed that BMI, ALT, AST, TG, HDL-C, SMI, CT_Liver_, and CT_L/S_ were significantly different in the distribution between participants with and without MASH in univariate analysis. After binary logistic regression, ALT, HDL-C, and CT_Liver_ were independent risk factors in predicting MASH (OR = 1.011, 95% CI = 1.002–1.019, *p* = 0.013; OR = 0.209, 95% CI = 0.060–0.722, *p* = 0.013; OR = 0.944, 95% CI = 0.924–0.964, *p* < 0.001, respectively).

**Table 1:** Comparison of clinical characteristics and CT-derived parameters between participants with non-MASH and MASH

Variables	non-MASH	MASH		Multivariable analysis	
	*N* = 138	*N* = 147	*p*	OR (95% CI)	*p*
Age, year	31 (13.0)	31 (10.0)	0.511		
Gender			0.214		
Male	42 (14.7)	55 (19.3)			
Female	96 (33.7)	92 (32.3)			
BMI, kg/m^2^	36.4 (9.9)	38.9 (7.6)	**0.019**	1.006 (0.950–1.065)	0.834
Hypertension			0.780		
Absence	102 (35.8)	101 (35.4)			
Presence	36 (12.6)	46 (16.1)			
AGM			0.271		
Absence	84 (29.5)	80 (28.1)			
Presence	54 (18.9)	67 (23.5)			
PLT, 10^9^/L	263 (76.0)	268 (69.0)	0.952		
ALT, U/L	29.6 (27.7)	53.4 (45.8)	**< 0.001**	1.011 (1.002–1.019)	**0.013**
AST, U/L	19.9 (11.4)	31.0 (25.9)	**< 0.001**	0.993 (0.974–1.013)	0.506
TBil, umol/L	10.4 (5.4)	10.2 (5.5)	0.786		
DBil, umol/L	1.7 (1.0)	1.9 (1.3)	0.092		
FBG, mmol/L	4.9 (1.1)	5.1 (1.6)	0.187		
TG, mmol/L	1.4 (1.0)	1.8 (1.4)	**< 0.001**	1.058 (0.912–1.228)	0.456
TC, mmol/L	4.8 (1.2)	4.7 (1.3)	0.343		
HDL-C, mmol/L	1.0 (0.4)	1.0 (0.2)	**0.001**	0.209 (0.060–0.722)	**0.013**
LDL-C, mmol/L	2.9 (1.0)	3.0 (1.2)	0.287		
CRP, mg/L	5.6 (7.0)	6.7 (6.1)	0.096		
SMI, cm^2^/m^2^	48.0 (11.7)	48.9 (10.7)	**0.038**	0.997 (0.965–1.030)	0.856
CT_Liver_, HU	46.9 (21.7)	30.2 (13.8)	**< 0.001**	0.944 (0.924–0.964)	**< 0.001**
CT_L/S_	1.0 (0.5)	0.6 (0.3)	**< 0.001**	0.377 (0.010-13.668)	0.594

Data are presented as median (interquartile) or *n* (%)

*MASH* metabolic dysfunction-associated steatohepatitis, *BMI* body mass index, *AGM* abnormal glucose metabolism, *PLT* Platelet, *ALT* alanine aminotransferase, *AST* aspartate aminotransferase, *TBil* total bilirubin, DBil direct bilirubin, *TG* triglyceride, *TC* total cholesterol, *FBG* fasting blood glucose, *HDL-C* high-density lipoprotein cholesterol, *LDL-C* low-density lipoprotein cholesterol, *CRP C*-reactive protein, *SMI* skeletal muscle index, *CT*_Liver_ liver CT attenuation value, *HU* Hounsfield unit, *CT*_L/S_ liver to spleen *CT* attenuation ratio, *OR* Odds Ratio

Boldface values indicate statistically significant results (*P* < 0.05)

#### Steatosis

Supplementary File 2 showed that multiple clinical characteristics and CT-derived parameters were significantly different in the distribution between different hepatic steatosis grade groups in univariate analysis. Binary logistic regression showed that ALT and CT_Liver_ were independent risk factors in predicting hepatic steatosis grade ≥ S1 (OR = 1.047, 95% CI = 1.004–1.090, *p* = 0.030; OR = 0.816, 95% CI = 0.754–0.883, *p* < 0.001, respectively) and grade S3 (OR = 1.015, 95% CI = 1.006–1.023, *p* = 0.001; OR = 0.887, 95% CI = 0.860–0.916, *p* < 0.001, respectively). BMI (OR = 1.088, 95% CI = 1.007–1.176, *p* = 0.032), HDL-C (OR = 0.180, 95% CI = 0.036–0.891, *p* = 0.036), SMI (OR = 0.937, 95% CI = 0.891–0.985, *p* = 0.011), and CT_Liver_ (OR = 0.854, 95% CI = 0.821–0.889, *p* < 0.001) were the independent predictors of hepatic steatosis grade ≥ S2 (Table 2).

**Table 2:** Multivariable analysis for different hepatic steatosis grades

Variables	S0 vs. ≥ S1		S0-1 vs. ≥ S2		S0-2 vs. S3	
	OR (95% CI)	*p*	OR (95% CI)	*p*	OR (95% CI)	*p*
Age					0.989	0.605
					(0.947–1.032)	
Gender	1.142	0.905	0.502	0.168		
	(0.129 − 0.124)		(0.188–1.338)			
BMI	0.911	0.060	1.088	**0.032**	0.999	0.960
	(0.826–1.004)		(1.007–1.176)		(0.942–1.058)	
Hyperte-nsion	4.213	0.057				
	(0.960-18.496)					
AGM	0.457	0.304	1.712	0.134		
	(0.102–2.038)		(0.848–3.455)			
PLT						
ALT	1.047	**0.030**	1.012	0.056	1.015	**0.001**
	(1.004–1.090)		(1.000-1.025)		(1.006–1.023)	
AST	0.942	0.393	0.986	0.317	0.990	0.340
	(0.820–1.081)		(0.958–1.014)		(0.969–1.011)	
TBil	0.966	0.594				
	(0.849–1.098)					
DBil	1.477	0.493	0.824	0.230	0.859	0.351
	(0.485–4.498)		(0.600-1.131)		(0.625–1.182)	
FBG	1.704	0.170	0.849	0.205	1.106	0.321
	(0.796–3.646)		(0.659–1.094)		(0.906–1.351)	
TG	0.927	0.833	1.061	0.494		
	(0.457–1.881)		(0.898–1.256)			
TC	1.301	0.917				
	(0.009-179.751)					
HDL-C	0.332	0.272	0.180	**0.036**		
	(0.046–2.375)		(0.036–0.891)			
LDL-C	0.948	0.910	1.567	0.070		
	(0.380–2.364)		(0.963–2.549)			
CRP			0.991	0.542	1.004	0.775
			(0.962–1.021)		(0.974–1.035)	
SMI	0.967	0.533	0.937	**0.011**		
	(0.869–1.075)		(0.891–0.985)			
CT_Liver_	0.816	**< 0.001**	0.854	**< 0.001**	0.887	**< 0.001**
	(0.754–0.883)		(0.821–0.889)		(0.860–0.916)	
CT_L/S_	0.058	0.274	0.033	0.143	0.270	0.631
	(0.000-9.589)		(0.000-3.153)		(0.001–56.211)	

*BMI* body mass index, *AGM* abnormal glucose metabolism, *PLT* Platelet, *ALT* alanine aminotransferase, *AST* aspartate aminotransferase, *TBil* total bilirubin, *DBil* direct bilirubin, *TG* triglyceride, *TC* total cholesterol, *FBG* fasting blood glucose, *HDL-C* high-density lipoprotein cholesterol, *LDL-C* low-density lipoprotein cholesterol, *CRP *C-reactive protein, *SMI* skeletal muscle index, *CT*_Liver_ liver CT attenuation value, *HU* Hounsfield unit, *CT*_L/S_ liver to spleen CT attenuation ratio, *OR* Odds Ratio

Boldface values indicate statistically significant results (*P* < 0.05)

### Diagnostic performance of combination models for predicting MASH and steatosis

#### MASH

**Fig. 3:**
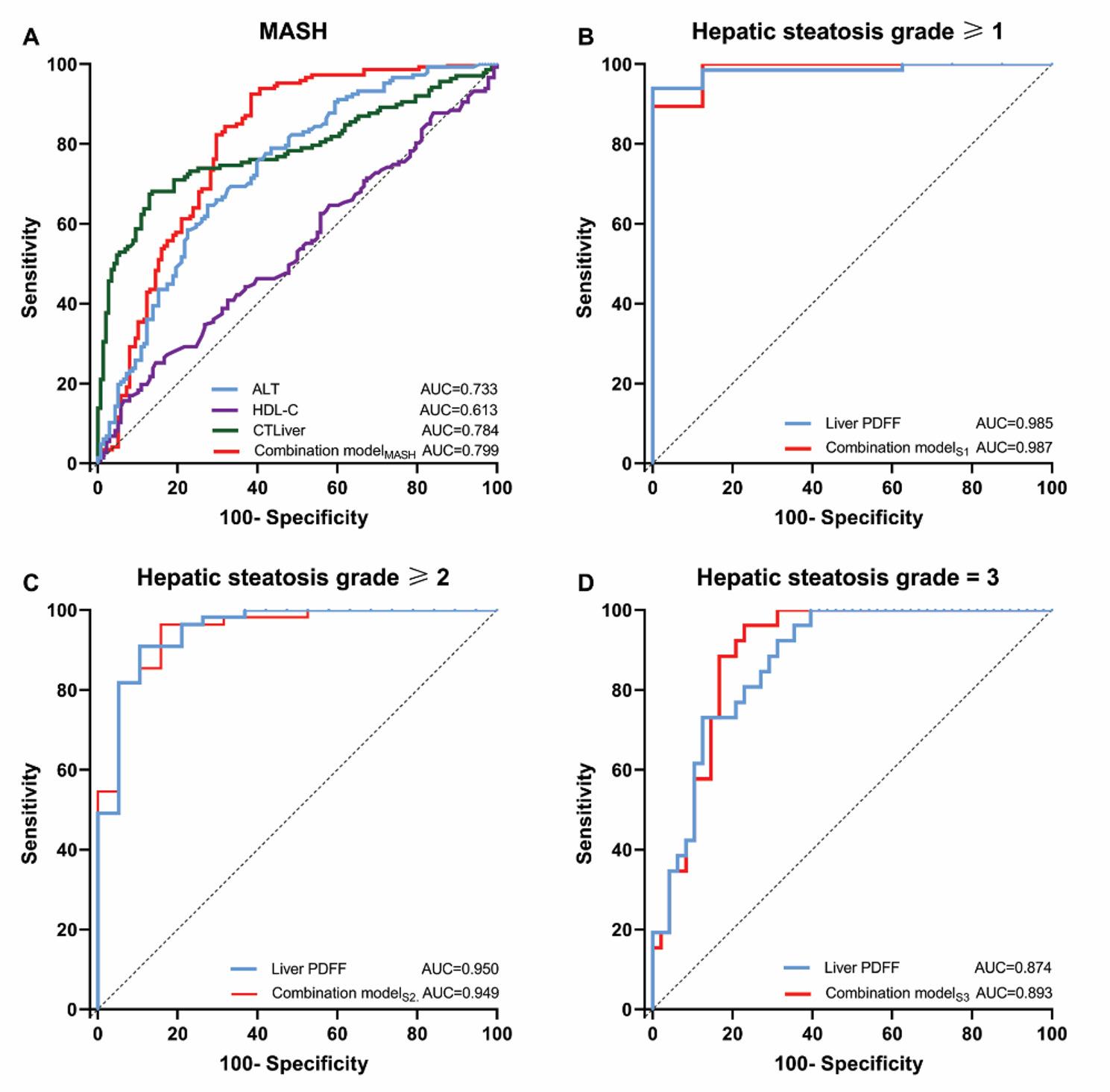
ROC curves of different combination models and liver PDFF in predicting MASH (**A**), hepatic steatosis grade ≥ S1 (**B**), grade ≥ S2 (**C**), and grade S3 (**D**). ALT, alanine aminotransferase. HDL-C, high-density lipoprotein cholesterol. CT_Liver_, liver CT attenuation value. PDFF, proton density fat fraction. Combination model_MASH_, a model combining ALT, HDL-C, and CT_Liver_. Combination model_S1_, a model combining ALT and CT_Liver_. Combination model_S2_, a model combining body mass index (BMI), HDL-C, skeletal muscle index (SMI), and CT_Liver_. Combination model_S3_, a model combining ALT and CT_Liver_.

**Fig. 4:**
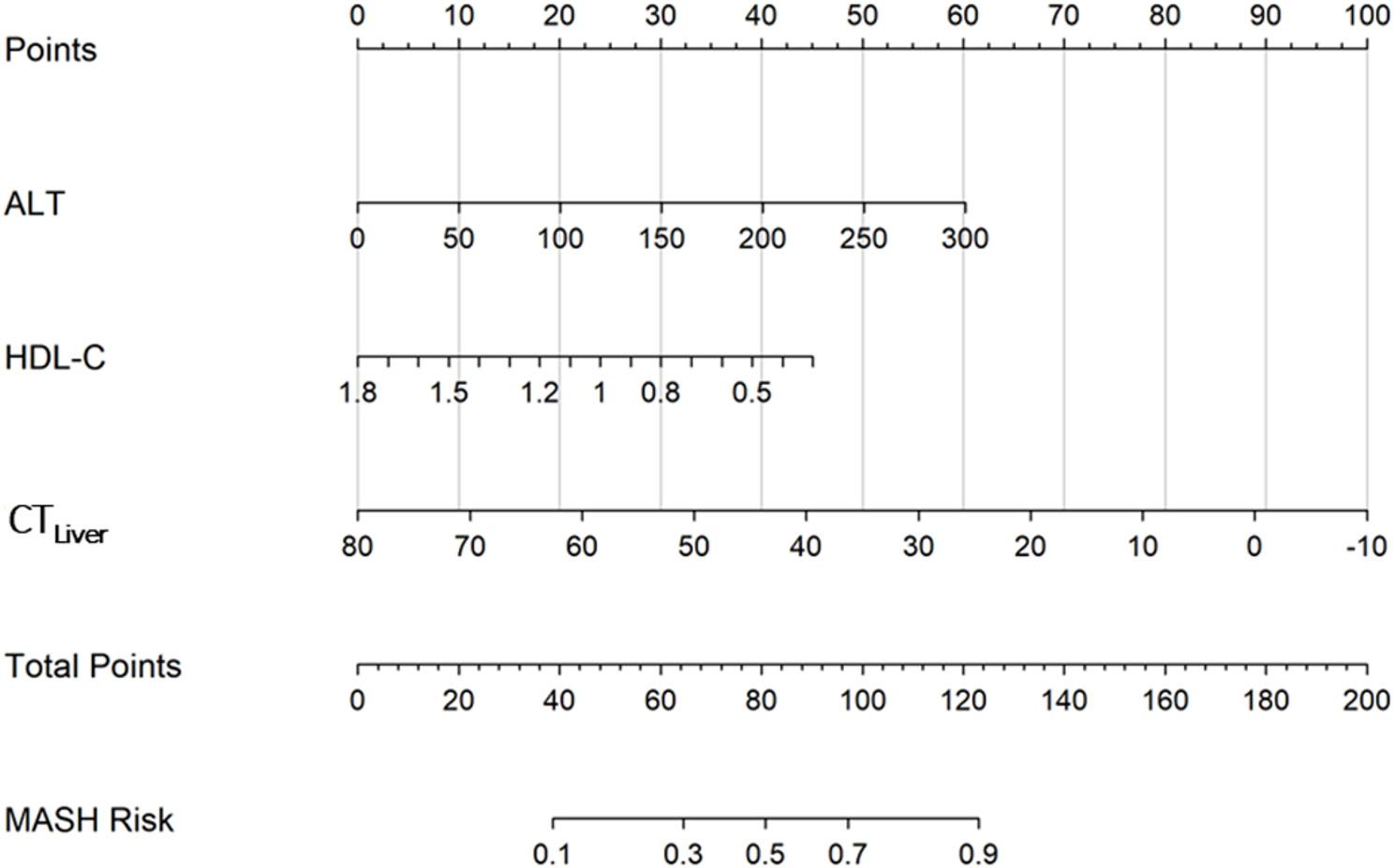
A nomogram for predicting MASH risk in patients with obesity prior to bariatric surgery

The ALT, HDL-C, and CT_Liver_ showed good diagnostic performance of differentiating MASH from non-MASH patients (AUC = 0.733, 0.613, and 0.784, respectively). The combination model_MASH_ demonstrated the best performance for identifying MASH with an AUC (95% CI) of 0.799 (0.748–0.844) (Table 3) (Fig. 3A). Based on this model, a nomogram was developed (Fig. 4). According to the nomogram, the corresponding values of each risk variable could be scored. The total score obtained by adding up the scores of each variable could be used to estimate the probability of MASH occurrence.

**Table 3:** Receiver operating characteristic curve analysis of MASH and hepatic steatosis grade

Variables	AUC (95% CI)	Cutoff value	Sen. (%)	Spe. (%)	PPV (%)	NPV (%)
MASH						
ALT, U/L	0.733 (0.677–0.783)	40.5	64.6	72.5	71.4	65.8
HDL-C, mmol/L	0.613 (0.554–0.670)	1.1	73.5	47.8	60.0	62.8
CT_Liver_, HU	0.784 (0.732–0.830)	41.3	86.4	68.1	74.3	82.4
Combination model_MASH_	0.799 (0.748–0.844)	0.4	92.5	61.6	72.0	88.5
Hepatic Steatosis grade						
Combination model_S1_	0.954 (0.924–0.974)	0.9	87.9	96.9	99.6	47.1
Combination model_S2_	0.927 (0.893–0.953)	0.6	86.2	84.8	90.7	78.2
Combination model_S3_	0.894 (0.855–0.925)	0.2	87.5	75.5	57.4	94.1

*MASH* metabolic dysfunction-associated steatohepatitis, *ALT* alanine aminotransferase, *HDL-C* high-density lipoprotein cholesterol, *CT*_Liver_ liver CT attenuation value, *HU* Hounsfield unit, *AUC* area under the curve, *Sen* Sensitivity, *Spe* Specificity, *PPV* positive predictive value, *NPV* negative predictive value

Combination model_MASH_, a model combining ALT, HDL-C, and CT_Liver_

Combination model_S1_, a model combining ALT and CT_Liver_

Combination model_S2_, a model combining BMI, HDL-C, SMI, and CT_Liver_

Combination model_S3_, a model combining ALT and CT_Liver_

#### Steatosis

The combination model_S1_ (ALT and CT_Liver_), combination model_S2_ (BMI, HDL-C, SMI, and CT_Liver_), and combination model_S3_ (ALT and CT_Liver_) showed good performance for predicting hepatic steatosis grade ≥ S1, ≥ S2, and grade S3 with an AUC (95% CI) of 0.954 (0.924–0.974), 0.927 (0.893–0.953), and 0.894 (0.855–0.925), respectively (Table 3).

### Comparison of diagnostic efficacy of different combination models and liver PDFF for predicting hepatic steatosis in a subgroup

This subgroup analysis included 74 participants who simultaneously underwent non-contrast chest CT and liver MRI-PDFF. For detecting hepatic steatosis grade ≥ S1, the AUC of the combination model_S1_ and liver PDFF was 0.987 (95% CI: 0.927–1.000) and 0.985 (95% CI: 0.924–0.999) (*p* = 0.912, DeLong’s test), respectively. For detecting hepatic steatosis of grade ≥ S2, the AUCs of the combination model_S2_ and liver PDFF were 0.949 (95% CI: 0.872–0.987) and 0.950 (95% CI: 0.873–0.987) (*p* = 0.946, DeLong’s test), respectively. For detecting hepatic steatosis of grade S3, the AUCs of the combination model_S3_ and liver PDFF were 0.893 (95% CI: 0.800–0.953) and 0.874 (95% CI: 0.777–0.940) (*p* = 0.430, DeLong’s test), respectively (Fig. 3B-D).

### Correlations between CT-derived parameters and hepatic steatosis grade, NAS

The CT_Liver_ and CT_L/S_ were significantly negatively correlated with the hepatic steatosis grade (*r* = -0.77 and - 0.76, both *p* < 0.001) and NAS (*r* = -0.69 and - 0.68, both *p* < 0.001), while SMI was significantly positively correlated with hepatic steatosis grade (*r* = 0.12, *p* = 0.031) and NAS (*r* = 0.18, *p* = 0.002).

### Interobserver agreement of liver PDFF, CT liver and spleen attenuation value

There was a perfect interobserver agreement for liver PDFF, CT liver and spleen attenuation value (ICC = 0.999, 95% CI = 0.998–1.000, ICC = 0.998, 95% CI = 0.995–0.999, ICC = 0.924, 95% CI = 0.847–0.963, respectively).

## Discussion

Over the past decades, MASLD has become a growing public health problem around the world. Exploring a widely accessible and non-invasive assessment tool is crucial for the clinical diagnosis of MASLD. Obesity has been considered as a significant risk factor that promotes the progression of MASLD [25]. Focusing on people with obesity undergoing bariatric surgery, we found that ALT, HDL-C, and CT_Liver_ were identified as independent risk factors for predicting MASH. The combination models of clinical characteristics and CT-derived parameters demonstrated favorable diagnostic performance in predicting MASH and hepatic steatosis grade, with AUCs ranging from 0.799 to 0.954. In addition, CT_Liver_, CT_L/S_, and SMI showed significant correlations with both hepatic steatosis grades and NAS. Our research findings are expected to have important implications for the non-invasive assessment of MASLD disease progression in people with obesity.

MASH is not only associated with liver fat content, but also with other indicators that are relevant to metabolic syndrome. A recent study reported that patients with MASLD have a higher ALT and lower HDL-C level compared to those without MASLD [26]. In our study, ALT and HDL-C were also independently associated with MASH. Our findings further indicated that the prediction model integrating ALT, HDL-C, and CT_Liver_ exhibits higher accuracy (AUC, 0.799) in diagnosing MASH compared to the individual predictive performance (AUCs, 0.613–0.784) of ALT, HDL-C or CT_Liver_ alone. A recent systematic review has researched the accuracy of non-invasive imaging techniques for diagnosing MASH in patients with MASLD, and five CT-related studies were enrolled with a range of AUCs from < 0.60 to 0.94 [27]. The differences in the target population, the definition of MASH, and the selection of CT parameters might be the reasons for the inconsistency between our research results and those of the above-mentioned studies. Our findings suggested that the clinical-CT combination model might offer clinical utility for MASH screening in people with obesity.

Non-contrast CT imaging plays an important role in the opportunistic detection of hepatic steatosis. Our results showed that CT_Liver_ and CT_L/S_ were significantly negatively correlated with the hepatic steatosis grade, which was consistent with previous research findings [21, 28]. It was primarily because the X-ray attenuation of triglycerides is lower than that of normal liver [29]. A recent study demonstrated that non-contrast CT is a reliable method for detecting at least moderate hepatic steatosis, whereas the supporting evidence for contrast-enhanced CT and dual-energy CT is insufficient to draw strong evidence-based conclusions [19]. Guo Z et al. further reported that quantitative CT-based liver fat showed good correlation and accuracy with liver MRI-PDFF [30].

MRI-PDFF is currently recognized as the most accurate non-invasive reference standard for the quantitative assessment of hepatic steatosis, providing precise quantification of liver fat content. However, it has limited clinical accessibility, and is not included in the routine preoperative assessment for patients undergoing bariatric surgery. Based on more widely accessible non-contrast chest CT, we developed three simple models that combine clinical features with CT-derived parameters to non-invasively predict hepatic steatosis grade. In subgroup analyses, the combination model_S1_ (ALT and CT_Liver_), combination model_S2_ (BMI, HDL-C, SMI, and CT_Liver_), and combination model_S3_ (ALT and CT_Liver_) all exhibited high sensitivity and specificity in detecting hepatic steatosis grade ≥ S1, ≥ S2, and S3, respectively, with diagnostic performance comparable to that of MRI-PDFF (AUC: 0.987, 0.949, and 0.893 vs. 0.985, 0.950, and 0.874, respectively). Our findings confirm that liver CT-based composite biomarker can serve as a convenient, accurate, and non-invasive screening tool for grading hepatic steatosis in patients with obesity, particularly when MRI-PDFF is unavailable.

A recent retrospective study utilizing two large-sample databases demonstrated that 94.71% and 99% of NAFLD cases met the MASLD diagnostic criteria, respectively, suggesting that current NAFLD evidence is broadly applicable to MASLD [31]. As a systemic disease affecting the whole body, obesity can promote deposition of adipose tissue outside the liver, leading to altered body composition including the SMI. It is worth noting that in our study, SMI was weakly but positively correlated with NAS. Wan Q et al. also reported a significant positive correlation between SMI and NAS in people with obesity, regardless of gender [32]. Similarly, SMI was also significantly higher in patients with liver biopsy-proven MASH [33]. However, in our study, multivariate analysis revealed that SMI had no statistically significant effect on the occurrence of liver biopsy-proven MASH. Similarly, a recent study also reported that there was no significant correlation between SMI and the occurrence of MASH [34]. Considering the differences in the diagnosis of MASH, the measurement methods of SMI and the characteristics of the study population according to the above studies, a larger sample study is needed to determine the detailed association between the SMI at T11 or T12 level and MASLD progression.

In our study, chest CT, the routine screening modality for cardiovascular and lung diseases prior to bariatric surgery, provides additional hepatic imaging information via its intrinsic scan coverage that encompasses a portion of the liver, eliminating the need for abdominal CT and its associated radiation exposure and costs. Moreover, preoperative abdominal CT is not recommended for bariatric surgery, given the lack of supporting evidence for its utility in the routine preprocedural planning [35].

There are several limitations in our study. First, this was a single-center retrospective study enrolling only patients with morbid obesity undergoing bariatric surgery. Accordingly, future studies should incorporate multicenter datasets to externally validate our findings, and further prospectively evaluate the diagnostic performance of our clinical prediction models for assessing hepatic histopathological changes following bariatric surgery. Second, while we standardized the tube voltage to 120 kV (the most widely used setting for clinical chest CT) to minimize minor variability in CT attenuation measurements introduced by scanners from different manufacturers, the diagnostic performance of our clinical prediction models still requires further validation at non-120 kV tube voltage to expand the generalizability of our findings. Finally, due to the limited anatomical coverage of non-contrast chest CT, SMI was measured at the T11/T12 vertebral level in our study, rather than the L3 vertebral level widely adopted in prior studies [36, 37]. Therefore, the correlation between CT-derived SMI and hepatic histopathological features in obese patients requires further validation in a larger cohort.

In conclusion, in this retrospective single-center study of morbidly obese patients undergoing bariatric surgery, we developed a potentially convenient and non-invasive method for identifying MASH and grading hepatic steatosis based on preoperative 120 kV non‑contrast chest CT. The liver CT-based composite biomarkers can also be used for patients who do not need bariatric surgery for early diagnosis and clinical stratified management of hepatic steatosis and/or MASH. Meanwhile, it also holds promise for non-invasive evaluation of the efficacy of bariatric surgery in MASH, which will be the goal for our future research.

## Supplementary Information

Supplementary Material 1.

Supplementary Material 2.

## Data Availability

The data in the current study are available from the corresponding author on reasonable request.

## Abbreviations

MASLD: Metabolic dysfunction-associated steatotic liver disease
MASH: Metabolic dysfunction-associated steatohepatitis
NAFLD: Non-alcoholic fatty liver disease
NAS: NAFLD activity score
HCC: Hepatocellular carcinoma
MRI-PDFF: Magnetic resonance imaging proton density fat fraction
FACT: Fat analysis calculation technique
CT: Computed tomography
SMI: Skeletal muscle index
CT_Liver_: Liver CT attenuation value
CT_L/S_: Liver to spleen CT attenuation ratio
kV: Kilovolts
BMI: Body mass index
HU: Hounsfield unit
AUC: Area under the curve
ROI: Region of interest
ROC: Receiver operating characteristic
ALT: Alanine aminotransferase
AST: Aspartate aminotransferase
HDL-C: High-density lipoprotein cholesterol
AGM: Abnormal glucose metabolism
PLT: Platelet
TBil: Total bilirubin
DBil: Direct bilirubin
TG: Triglycerides
TC: Total cholesterol
LDL-C: Low-density lipoprotein cholesterol
CRP: C-reactive protein
