# Supplementary material for: Liver CT-based composite biomarkers can identify MASH and steatosis grade in people with obesity prior to bariatric surgery: a retrospective study
Source: BMC Gastroenterol. 2026 May 14;26:419. doi: 10.1186/s12876-026-04914-2 (PMC13339996; doi:10.1186/s12876-026-04914-2)
Supplement: Supplementary file 1 — Supplementary Material 1. [file 12876_2026_4914_MOESM1_ESM.docx]

**Supplementary 1**. Comparison of p values of clinical-CT parameters of steatosis and MASH in different groups with Shapiro-Wilk test for normality assumption

| Variables | Hepatic steatosis grade | | | | | | MASH | |
| --- | --- | --- | --- | --- | --- | --- | --- | --- |
|  | S0 vs. ≥ S1 | | S0-1 vs. ≥ S2 | | S0-2 vs. S3 | | non-MASH vs. MASH | |
| Age | 0.238 | < 0.001 | 0.015 | 0.003 | < 0.001 | 0.128 | 0.003 | 0.007 |
| BMI | 0.11 | < 0.001 | 0.001 | < 0.001 | < 0.001 | 0.299 | < 0.001 | 0.006 |
| PLT | 0.566 | < 0.001 | 0.047 | < 0.001 | 0.004 | < 0.001 | 0.009 | 0.001 |
| ALT | < 0.001 | < 0.001 | < 0.001 | < 0.001 | < 0.001 | < 0.001 | < 0.001 | < 0.001 |
| AST | 0.110 | < 0.001 | < 0.001 | < 0.001 | < 0.001 | < 0.001 | < 0.001 | < 0.001 |
| TBil | 0.428 | < 0.001 | < 0.001 | < 0.001 | < 0.001 | < 0.001 | < 0.001 | < 0.001 |
| DBil | 0.036 | < 0.001 | < 0.001 | < 0.001 | < 0.001 | 0.004 | < 0.001 | < 0.001 |
| FBG | 0.027 | < 0.001 | < 0.001 | < 0.001 | < 0.001 | < 0.001 | < 0.001 | < 0.001 |
| TG | < 0.001 | < 0.001 | < 0.001 | < 0.001 | < 0.001 | < 0.001 | < 0.001 | < 0.001 |
| TC | 0.095 | < 0.001 | 0.110 | < 0.001 | 0.217 | ＜0.001 | < 0.001 | < 0.001 |
| HDL-C | 0.024 | < 0.001 | < 0.001 | 0.009 | < 0.001 | 0.114 | < 0.001 | 0.028 |
| LDL-C | 0.035 | 0.003 | 0.864 | 0.006 | 0.324 | 0.028 | 0.023 | 0.146 |
| CRP | 0.003 | < 0.001 | < 0.001 | < 0.001 | < 0.001 | < 0.001 | < 0.001 | < 0.001 |
| SMI | 0.006 | < 0.001 | < 0.001 | < 0.001 | < 0.001 | 0.029 | < 0.001 | 0.001 |
| CT_Liver_ | < 0.001 | 0.014 | 0.055 | 0.008 | 0.097 | 0.034 | < 0.001 | 0.002 |
| CT_L/S_ | 0.005 | 0.004 | 0.726 | 0.002 | 0.639 | 0.030 | < 0.001 | 0.001 |

Abbreviations: PLT, Platelet. ALT, alanine aminotransferase. AST, aspartate aminotransferase. TBil, total bilirubin. DBil, direct bilirubin. TG, triglyceride. TC, total cholesterol. FBG, fasting blood glucose. HDL-C, high-density lipoprotein cholesterol. LDL-C, low-density lipoprotein cholesterol. CRP, C-reactive protein. SMI, skeletal muscle index. CT_Liver_, liver CT attenuation value. HU, Hounsfield units. CT_L/S_, liver to spleen CT attenuation ratio. p value < 0.05, which denies the normality assumption of the parameter.
