# Supplementary material for: Liver CT-based composite biomarkers can identify MASH and steatosis grade in people with obesity prior to bariatric surgery: a retrospective study
Source: BMC Gastroenterol. 2026 May 14;26:419. doi: 10.1186/s12876-026-04914-2 (PMC13339996; doi:10.1186/s12876-026-04914-2)
Supplement: Supplementary file 2 — Supplementary Material 2. [file 12876_2026_4914_MOESM2_ESM.docx]

**Supplementary 2.** Comparison of clinical characteristics and CT-derived parameters across different hepatic steatosis grades

| Variables | S0 vs. ≥ S1 | | | S0-1 vs. ≥ S2 | | | S0-2 vs. S3 | | |
| --- | --- | --- | --- | --- | --- | --- | --- | --- | --- |
|  | N = 32 | N = 289 | *p* | N = 118 | N = 203 | *p* | N = 233 | N = 88 | *p* |
| Age, year | 29.5 (15.3) | 31.0 (11.0) | 0.868 | 32.0 (12.3) | 30.0 (51.0) | 0.220 | 32.0 (11.0) | 29.0 (12.0) | 0.031 |
| Gender |  |  | 0.002 |  |  | 0.040 |  |  | 0.570 |
| Male | 3 (0.9) | 107 (33.3) |  | 32 (10.0) | 78 (24.3) |  | 82 (25.5) | 28 (8.7) |  |
| Female | 29 (9.0) | 182 (56.7) |  | 86 (26.8) | 125 (38.9) |  | 151 (47.0) | 60 (18.7) |  |
| BMI, kg/m^2^ | 34.9 (7.0) | 37.9 (8.1) | 0.019 | 35.6 (7.8) | 39.0 (8.3) | <0.001 | 37.2 (8.4) | 39.0 (7.8) | 0.033 |
| Hypertension |  |  | 0.009 |  |  | 0.693 |  |  | 0.627 |
| Absence | 29 (9.0) | 198 (61.7) |  | 85 (26.5) | 142 (44.2) |  | 163 (50.8) | 64 (19.9) |  |
| Presence | 3 (0.9) | 91 (28.3) |  | 33 (10.3) | 61 (19.0) |  | 70 (21.8) | 24 (7.5) |  |
| AGM |  |  | 0.010 |  |  | 0.034 |  |  | 0.465 |
| Absence | 25 (7.8) | 157 (48.9) |  | 76 (23.7) | 106 (33.0) |  | 135 (42.1) | 47 (14.6) |  |
| Presence | 7 (2.2) | 132 (41.1) |  | 42 (13.1) | 97 (30.2) |  | 98 (30.5) | 41 (12.8) |  |
| PLT, 10^9^/L | 281.5 (134.0) | 264.0 (71.0) | 0.341 | 263.0 (93.8) | 269.0 (65.0) | 0.626 | 271.0 (82.5) | 258.5 (63.8) | 0.411 |
| ALT, U/L | 16.2 (8.3) | 40.1 (40.4) | <0.001 | 26.1 (21.4) | 49.4 (44.3) | <0.001 | 33.7 (30.6) | 65.0 (55.4) | <0.001 |
| AST, U/L | 16.2 (2.8) | 25.8 (19.7) | <0.001 | 18.6 (8.9) | 30.2 (24.3) | <0.001 | 20.8 (14.0) | 35.7 (31.4) | <0.001 |
| TBil, umol/L | 9.0 (4.5) | 10.4 (5.6) | 0.035 | 10.2 (5.5) | 10.2 (5.3) | 0.645 | 10.3 (5.5) | 10.0 (5.0) | 0.869 |
| DBil, umol/L | 1.4 (0.7) | 1.9 (1.2) | <0.001 | 1.6 (1.0) | 1.9 (1.3) | 0.006 | 1.7 (1.1) | 2.1 (1.2) | 0.028 |
| FBG, mmol/L | 4.6 (0.7) | 5.1 (1.6) | 0.002 | 4.8 (1.1) | 5.2 (1.8) | 0.005 | 4.9 (1.3) | 5.4 (1.8) | 0.014 |
| TG, mmol/L | 1.3 (0.8) | 1.7 (1.2) | <0.001 | 1.4 (1.0) | 1.7 (1.3) | 0.001 | 1.5 (1.1) | 1.7 (1.4) | 0.151 |
| TC, mmol/L | 4.3 (1.5) | 4.8 (1.1) | 0.028 | 4.8 (1.2) | 4.7 (1.2) | 0.161 | 4.7 (1.1) | 4.8 (1.5) | 0.188 |
| HDL-C, mmol/L | 1.1 (0.5) | 1.0 (0.3) | 0.049 | 1.1 (0.4) | 1.0 (0.3) | 0.001 | 1.0 (0.3) | 1.0 (0.3) | 0.823 |
| LDL-C, mmol/L | 2.6 (1.2) | 3.0 (1.0) | 0.038 | 2.8 (1.0) | 3.0 (1.1) | 0.014 | 2.9 (1.0) | 3.1 (1.4) | 0.083 |
| CRP, mg/L | 5.5 (6.0) | 6.3 (7.1) | 0.268 | 4.9 (6.0) | 6.8 (6.9) | 0.001 | 5.6 (6.2) | 7.5 (7.3) | 0.002 |
| SMI, cm^2^/m^2^ | 43.9 (10.3) | 48.7 (11.4) | 0.001 | 45.7 (12.7) | 48.8 (10.5) | 0.024 | 48.0 (12.0) | 48.6 (10.2) | 0.657 |
| CT_Liver_, HU | 60.3 (7.4) | 34.6 (18.9) | <0.001 | 51.6 (14.5) | 29.3 (15.1) | <0.001 | 43.5 (20.3) | 23.5 (19.5) | <0.001 |
| CT_L/S_ | 1.2 (0.2) | 0.7 (0.4) | <0.001 | 1.0 (0.3) | 0.6 (0.3) | <0.001 | 0.9 (0.4) | 0.5 (0.4) | <0.001 |

Data are presented as median (interquartile) or n (%). MASH, metabolic dysfunction-associated steatohepatitis. BMI, body mass index. AGM, abnormal glucose metabolism. PLT, Platelet. ALT, alanine aminotransferase. AST, aspartate aminotransferase. TBil, total bilirubin. DBil, direct bilirubin. TG, triglyceride. TC, total cholesterol. FBG, fasting blood glucose. HDL-C, high-density lipoprotein cholesterol. LDL-C, low-density lipoprotein cholesterol. CRP, C-reactive protein. SMI, skeletal muscle index. CT_Liver_, liver CT attenuation value. HU, Hounsfield units. CT_L/S_, liver to spleen CT attenuation ratio. OR, Odds Ratio.
